# Are Pediatric Cancer Patients a Risk Group for Vitamin D Deficiency? A Systematic Review

**DOI:** 10.3390/cancers16244201

**Published:** 2024-12-17

**Authors:** Alexandru Alexandru, Cristiana-Smaranda Ivan, Sonia Tanasescu, Licina Andrada Oprisoni, Tiberiu-Liviu Dragomir, Norberth-Istvan Varga, Diana Mateescu, Mircea Diaconu, Madalin-Marius Margan, Estera Boeriu

**Affiliations:** 1 Department of General Medicine, “Victor Babes” University of Medicine and Pharmacy, Eftimie Murgu Square 2, 300041 Timisoara, Romania; alexandru.alexandru@student.umft.ro (A.A.); smaranda.ivan@student.umft.ro (C.-S.I.); 2 Department of Pediatrics, “Victor Babes” University of Medicine and Pharmacy, Eftimie Murgu Square 2, 300041 Timisoara, Romania; oprisoni.licinia@umft.ro (L.A.O.); estera.boeriu@umft.ro (E.B.); 3 Medical Semiology II Discipline, Internal Medicine Department, “Victor Babes” University of Medicine and Pharmacy, Eftimie Murgu Square 2, 300041 Timisoara, Romania; dragomir.tiberiu@umft.ro; 4 Doctoral School, Department of General Medicine, “Victor Babes” University of Medicine and Pharmacy, Eftimie Murgu Square 2, 300041 Timisoara, Romania; norberth.varga@umft.ro (N.-I.V.); diana.mateescu@umft.ro (D.M.); 5 Department of Obstetrics and Gynecology, Faculty of Medicine, “Victor Babes” University of Medicine and Pharmacy, Eftimie Murgu Square 2, 300041 Timisoara, Romania; diaconu.mircea@umft.ro; 6 Department of Functional Sciences, Discipline of Public Health, “Victor Babes” University of Medicine, and Pharmacy, Eftimie Murgu Square 2, 300041 Timisoara, Romania; margan.madalin@umft.ro

**Keywords:** 25(OH)D, vitamin D, vitamin D deficiency, pediatric, children, cancer, acute lymphoblastic leukemia, acute myeloid leukemia, solid tumors

## Abstract

**Background**: Vitamin D deficiency is increasingly recognized as a global health concern, with potential implications for cancer development and progression. This systematic review investigated the prevalence of vitamin D deficiency in pediatric cancer patients and its potential impact on clinical outcomes. **Methods**: A comprehensive literature search was conducted across multiple databases, including PubMed, Web of Science, and Cochrane Library, to identify the relevant studies published between 2009 and July 2024. Studies were included if they assessed vitamin D status in pediatric cancer patients and reported on the clinical outcomes. Data extraction and quality assessment were performed independently by two reviewers. **Results**: The review included 20 original articles encompassing a diverse pediatric population with various cancer types. A high prevalence of vitamin D deficiency was observed across the studies. Deficiency was associated with older age and lower socioeconomic status. Several studies reported associations between vitamin D deficiency and the increased risk of infection, poorer treatment response, and decreased survival. **Conclusions**: Vitamin D deficiency is highly prevalent in pediatric cancer patients and may negatively impact clinical outcomes. Routine screening for vitamin D deficiency and personalized supplementation strategies should be considered in this population. Further research is needed to establish optimal vitamin D management protocols and evaluate the long-term benefits of vitamin D repletion in pediatric oncology.

## Simple Summary

Vitamin D plays a vital role in supporting healthy growth and development. Deficiency in this nutrient has been associated with various health problems, including an increased susceptibility to infections and other complications, particularly in vulnerable populations. Pediatric cancer patients are at a greater risk of vitamin D deficiency due to combined effects of illness, treatments, days of hospitalization, and ongoing developmental needs. This study reviewed the medical literature to understand how common vitamin D deficiency is in this type of patient and its potential impact on treatment outcomes, trying to conclude whether they are a risk group for vitamin D impairment. These findings highlight the importance of checking vitamin D levels at diagnosis and throughout treatment, alongside providing supplements when needed, to support better treatment responses and overall health in pediatric oncology patients.

## 1. Introduction

Vitamin D, a fat-soluble prohormone, is best known for its critical role in maintaining calcium and phosphate homeostasis and affects bone mineralization and remodeling while also extending beyond generic roles [1,2,3,4]. This essential nutrient exists in two major forms: vitamin D2 (ergocalciferol), primarily obtained from dietary sources such as fortified foods and supplements, and vitamin D3 (cholecalciferol), synthesized in the skin upon exposure to ultraviolet B (UVB) radiation [5]. Both forms undergo metabolic activation in the liver and kidneys to become the biologically active form, 1,25-dihydroxyvitamin D (calcitriol) [1,2,3,4,5]. However, extrarenal processes of hydroxylation based on 25-hydroxyvitamin D-1α-hydroxylase present in various tissues are more important than classical metabolic pathways [6].

The vitamin D receptor (VDR) regulates gene expression involved in many physiological processes, including cell growth and differentiation, immune function through T and B lymphocytes, and inflammation, playing a crucial role in anti-cancer defense mechanisms [7,8,9,10,11,12,13,14,15]. VDR polymorphism plays an equally important role, with impact on cancer risk, evolution, and even prognosis value [16,17,18,19,20].

Furthermore, vitamin D also exhibits anti-inflammatory properties, reducing the production of pro-inflammatory cytokines and promoting the expression of anti-inflammatory mediators [8,9,10,21,22]. Noteworthy evidence suggests that there are many biochemical pathways involved in cancer pathogenesis, such as cell proliferation, apoptosis, angiogenesis, and immune response [23,24,25,26], inhibiting cell cycle progression and inducing apoptosis [27,28], with direct implications in various cancers such as osteosarcoma, prostate, colorectal, breast, etc. [6,11,29,30,31,32,33,34].

While these associations are well documented in the general adult population, homogeneity in results remains elusive [35,36], and the implication of vitamin D deficiency in pediatric cancer patients warrants specific attention.

For instance, children with sufficient 25(OH)D levels have demonstrated better survival rates post-hematopoietic stem cell transplantation (HSCT) compared to those who are deficient [37,38]. Adequate levels can potentially improve treatment outcomes and reduce complications such oral mucositis [39,40] or even graft-versus-host disease [38], however, with no reduction in incidence.

Previous research shows little quality evidence about the association between lower 25OHD levels and lower BMD Z-scores in children with cancer, the consensus being that better screening and supplementation are necessary [41].

The existing studies, while valuable, present diverse methodologies and patient populations, creating a need for a comprehensive synthesis of the available data [40,42,43,44,45,46,47,48,49,50,51,52,53,54,55,56,57,58,59,60]. The variability in response to supplementation is also worth noting not only in cancer but even other pathologies such as cardiovascular diseases [61,62].

Assessment of vitamin D status is typically determined by measuring serum concentrations of 25-hydroxyvitamin D [25(OH)D]. Different organizations and expert bodies have established varying thresholds for deficiency and insufficiency, being partly responsible for the variability of data. This is illustrated in Table 1 [63,64,65,66].

This systematic review aims to synthesize and critically evaluate the current literature on the vitamin D status in pediatric cancer patients. Our study has two primary objectives: to assess the prevalence of vitamin D deficiency among pediatric cancer patients and to synthetize the existing literature on the potential impact of vitamin D status and supplementation on clinical outcomes in pediatric oncology. By summarizing current data, this review seeks to highlight gaps in knowledge and guide future research in this important area.

## 2. Materials and Methods

### 2.1. Search Strategy

This study followed the Preferred Reporting Items for Systematic reviews and Meta-Analyses (PRISMA) guidelines [67]. A comprehensive literature search was conducted across multiple databases, including MDPI, PubMed, Web of Science, Science Direct, and the Cochrane Library. The search terms included “Vitamin D”, “pediatric cancer”, “ALL”, “Acute Lymphoblastic Leukemia”, “AML”, “Acute Myeloid Leukemia”, and “Central Nervous System Neoplasms”, within a time period from 2014 to 2024. This was performed to include papers that contain data of patients as close as possible to the present lifestyles and treatment protocols in current medical practice. The review included original research articles with the following study designs: randomized controlled trials, prospective and retrospective observational cohort studies, cross-sectional studies, and case-control studies.

### 2.2. Study Selection

**Figure 1:**
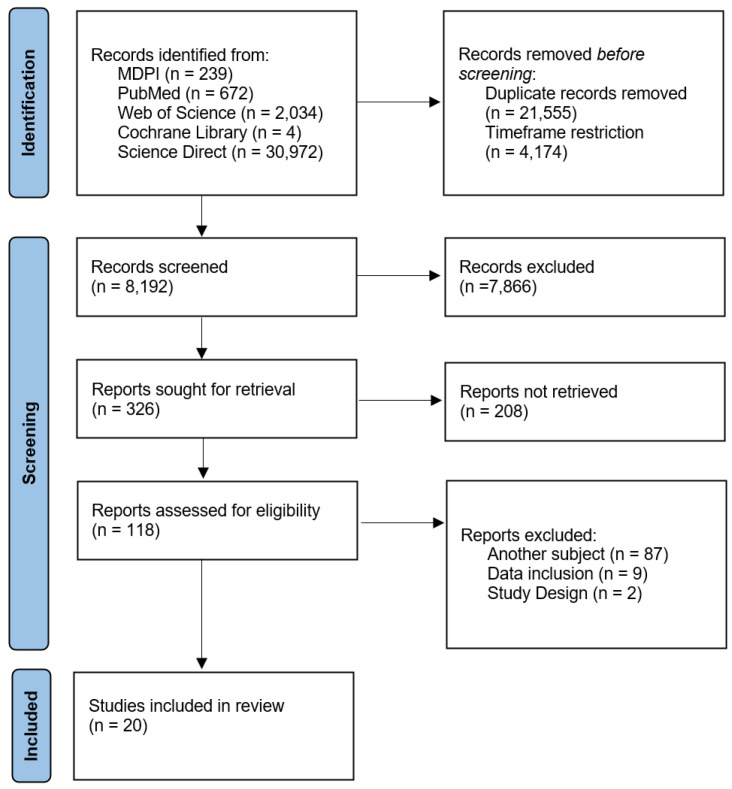
PRISMA flowchart of the study selection process.

This review included only full-length, peer-reviewed articles published in English that focused on the impact of vitamin D deficiency on pediatric cancer patients. The evaluation considered vitamin D deficiency rates, infection rates, bone health outcomes, immune function, and overall survival rates. Studies were excluded if they (1) did not involve pediatric cancer patients; (2) lacked data on clinical outcomes related to vitamin D status; (3) were not published in a peer-reviewed journal; (4) did not have an English translation available; or (5) used a systematic review/meta-analysis/scientific letter methodology. To maintain the reliability of the screening process, two independent reviewers, N.I.V. and D.M., screened all the records independently for eligibility. The inter-rater reliability obtained by Cohen’s Kappa value was 0.86, which signifies a very high degree of agreement. In the case of any discrepancies at this stage, they were resolved by either consensus or a third reviewer (E.B.). The study selection process, following the PRISMA guidelines, is illustrated in Figure 1.

### 2.3. Data Extraction

After the study selection process, all the included publications were independently reviewed by two evaluators (A.A. and C.S.I.). Data extraction was independently performed using a standardized form. Information was organized into tables that included authors’ names, year of publication, study design, mean age of study population, type of cancer, control and study groups, sex expressed as a percentage of male and female patients, and mean 25(OH)D levels. Any discrepancies were resolved through consensus with a third reviewer (E.B.).

For the purpose of this review, we used Pediatric Endocrine Society (Table 1) thresholds and mainly looked at the insufficiency (≤20 ng/mL) in 25(OH)D levels [64]. When the 25(OH)D levels of the study group were not actually present in the studies but were quantified under different descriptions, the term “Inadequacy” was used to describe values < 30 ng/mL, and the term “Sufficiency” for >30 ng/mL was used to describe the prevalence percentages depending on the available data in the original paper. This was performed to contain and present the variability of data in a more homogenous way.

For papers that contained more than one study group or more than one 25(OH)D measurement, the main interest group and first or preintervention measurement are presented in Table 2, to avoid cluttering and confusion.

Our study also adheres to the evidence-based recommendations for the presentation of numerical data to ensure clarity, precision, and reader accessibility. This results in the use of significant digits, decimal places, and context-specific rounding rules to appropriately balance accuracy and simplicity [68].

### 2.4. Quality Assessment

Two reviewers (L.A.O. and D.M.) independently conducted a quality assessment of the included articles, utilizing the National Institutes of Health Study Quality Assessment Tools, accessible at www.nhlbi.nih.gov/health-topics/study-quality-assessment-tools (accessed on 19 July 2024), and for the case report, the assessment tool utilized was the Checklist for Case Reports accessible at https://jbi.global/critical-appraisal-tools (accessed on 19 July 2024). If discrepancies arose, they were settled by holding a discussion with E.B. The results of the quality assessment, conducted using the NIH Quality Assessment Tool, are summarized in Supplemental Material, Table S1.

## 3. Results

### 3.1. Overview of the Selected Studies

Upon the initial search using different combinations and variants of keywords in advanced search, 33,921 studies were identified addressing vitamin D, various types of cancer, and different study populations (including non-pediatric patients) using MDPI, PubMed, Web of Science, Cochrane Library, and Science Direct databases. No time restrictions were initially applied, but after a 10-year timeframe was selected, 4174 articles were excluded, and 21,555 duplicates were removed, leaving 8192 studies for further analysis. Upon the first round of analysis, 326 were considered and went under a second round upon which the full texts of 118 studies were retrieved for a full-text assessment. After applying the exclusion criteria to the remaining articles, 20 were selected to be included in the review process. The study selection process is summarized in Figure 1.

This systematic review included studies on children and adolescents with various cancer types, predominantly hematologic malignancies such as acute lymphoblastic leukemia (ALL) and solid tumors. The median age across the included studies varied considerably, primarily focusing on children aged 0–15 years, with mean ages ranging from approximately 4 to 15 years. Several studies incorporated control groups of healthy children matched for age and gender. The reviewed studies also reflected the ethnic and socioeconomic diversity, with older children, non-Caucasian groups, and those from lower socioeconomic backgrounds exhibiting a higher prevalence of vitamin D deficiency. Table 2 offers an overview of the studies included in our review.

Across multiple studies, oncology pediatric patients consistently exhibited significantly lower 25(OH)D levels compared to the healthy controls [42,44,45,47,49,52].

Vitamin D inadequacy is highly prevalent among pediatric cancer patients, with rates ranging from 23% to 72% depending on the study and cancer type.

### 3.2. The Impact of Different Supplementation Regimens upon Vitamin D Levels

Five of the included studies contained different means of supplementation along with the obtained results, as summarized in Table 3.

Stoss therapy seems to be the most effective in rapidly achieving sufficiency, making it suitable for urgent correction, although maintenance dosing is required. Even though sufficiency was reported in 96% of the cases, the sufficiency levels declined over time, dropping to 35% by day 100 post-HSCT [44]. Orgel et al.’s [57] findings also showed the efficiency of high-dose supplementation for a median of 7 months of supplementation resulting in a mean 20 ng/mL 25(OH)D increase.

Bhandari et al. [43] conducted a non-randomized controlled trial, evaluating the impact of high-dose vitamin D supplementation (Stoss therapy) [69] versus standard oral supplementation [70] in patients undergoing hematopoietic stem cell transplantation (HSCT). The study group (SG, 29/33) had a mean 25(OH)D level increase from 28 ng/mL to 72 ng/mL within two weeks post-supplementation, with a later peak of 114 ng/mL. In comparison, a historical control cohort (HC, 15/136) receiving standard supplementation achieved lower rates of vitamin D sufficiency, with 97% of SG vs. 67% of HC reaching sufficient levels (*p* < 0.001). Despite initial success, sufficiency in the SG declined from 96% at day 0 to 35% by day 100. Limitations included the non-randomized design, small sample size, and variability in response.

The specific Stoss therapy dosing regimen [69] used in this study is detailed in Table 4.

Similarly, a 2017 randomized controlled trial by Orgel et al. [57] investigated the impact of High-Dose Supplementation after switching from vitamin D3 and calcium citrate supplementation in 49 adolescents (aged 10–21 years, median age of 15 years) with ALL. Initially designed as a double-blinded, placebo-controlled trial with daily oral supplementation, the study was amended to an open-label trial using Directly Observed Therapy (DOT) due to poor adherence. The DOT intervention group (*n* = 29) significantly increased serum 25(OH)D levels from 20 ± 5 ng/mL at baseline to 27 ± 12 ng/mL at study end (*p* = 0.03), while the standard of care (SOC) group showed no significant change (19 ± 4 ng/mL to 19 ± 7 ng/mL). However, the DOT group showed no preventive bone density decline, highlighting the need for additional strategies.

High-dose therapy proves useful prior to hematopoietic stem cell transplantation or other acute conditions, while weekly and daily regimens provided more gradual increases, with a possible better suitability for long-term management.

Juhász et al. [45] evaluated the vitamin D status in 173 children with solid tumors (mean age: 6 years), comparing them to healthy controls. The patient group had a mean baseline 25(OH)D level of 24 ng/mL, 6 ng/mL lower than the controls. Daily supplementation with 1000–2000 IU of vitamin D significantly increased serum levels, rising from 24 ng/mL to 28 ng/mL after 4–6 weeks (*p* = 0.02).

In cases of extreme deficiency, even low-dose daily cholecalciferol was highly effective in resolving the deficiency and associated complications.

Cook et al. [59] described a 4-month-old male infant with AML who presented with severe 25(OH)D deficiency (level of 6 ng/mL). The infant had been exclusively breastfed without vitamin D supplementation. Prompt administration of oral cholecalciferol (2000 IU) effectively corrected the deficiency and associated hypocalcemia. After 3 months of supplementation, the 25(OH)D level reached 97 ng/mL. Chemotherapy was initiated following the correction of serum calcium levels, and the patient achieved remission after two cycles. This case highlights the importance of assessing vitamin D status in pediatric AML patients, particularly when electrolyte imbalances are present.

In a retrospective cross-sectional study from 2020, Aristizabal et al. [44] administered oral vitamin D supplementation, implementing a non-standardized supplementation approach. Doses ranged from 3000–50,000 IU per week, based on the initial levels. After measuring the 25(OH)D level in 163 children that were newly diagnosed with cancer, the mean 25(OH)D level was 28 ng/mL, with only 36% of patients exhibiting sufficient levels. The mean increase in the 25(OH)D levels following supplementation was +12 ng/mL, with 68% of the supplemented patients achieving vitamin D sufficiency. Notably, among 115 patients who received vitamin D supplementation, those with solid tumors had mean 25(OH)D concentrations that were 7 ng/mL lower than patients with hematologic malignancies (*p* = 0.003).

Tailored approaches based on initial deficiency severity, cancer type, and patient adherence can be essential for optimal outcomes; however, clear supplementation protocols are needed to assure conciliation between findings.

### 3.3. Correlation Between Vitamin D Levels and Sociodemographic Parameters

This review highlights several risk factors for 25(OH)D impairment mentioned across multiple studies. The most notable findings are presented in Table 5.

Nine studies [40,42,44,48,49,55,56,58,60] identified a correlation between increasing age and vitamin D insufficiency in pediatric oncology patients. For instance, children over 10 years had a higher risk of deficiency with 25(OH)D being on average 4.6ng/mL lower (*p* = 0.019) [45]. Another study showed that age 6 and older had a 3.2-fold greater risk [50]. The deficiency rates increased from 23% in pre-school-aged children to 45% in school-aged children [58]. However, one study found no association between age and vitamin D levels [46].

Four studies [44,47,54,56] identified female sex as a risk factor for lower 25(OH)D levels. For instance, females demonstrated 25(OH)D levels that were 6 ng/mL lower than males (*p* = 0.005) [44]. Yet, two studies found no significant correlation between sex and lower vitamin D levels [46,60].

Two studies [42,49] found a significant correlation between race and vitamin D insufficiency, with African-American children showing the lowest 25(OH)D levels. Similarly, Hispanic ethnicity was identified as a risk factor for vitamin d insufficiency (*p* = 0.002) [44]. Two studies, however, reported no significant association between vitamin D and race [46,60].

Seasonal variations were reported inconsistently, while one study observed significantly higher levels during synthesizing periods (57 nmol/L vs. 26 nmol/L, *p* = 0.003) [6,48].

A study from 2019 conducted by Oosterom et al. [40] reported that vitamin D deficiency was observed in 8% of patients (<30 nmol/L) and 33% (<50 nmol/L) at T0, with a higher prevalence among children older than 4 years compared to those aged 1 to 4 years.

A 2018 longitudinal observational study conducted by Gokcebay et al. [46] evaluated the 25(OH)D level in 42 children (mean = 9 years) with lymphoma and various solid tumors during the first 6 months after diagnosis. At the time of presentation, the mean 25(OH)D level was 14 ng/mL (SD = ±8), with minimal variation observed at 3 months and 6 months (14 ± 7 ng/mL at 6 months). Vitamin D deficiency was prevalent in 79% of the patients. No significant differences in 25(OH)D levels were found based on sex, age (below or above 8 years), primary disease (lymphoma or solid tumor), season of assessment, or residence in an urban or rural area.

Iniesta et al. [48] evaluated plasma 25(OH)D levels in 65 pediatric oncology patients (median = 4 years) and 33 healthy controls (median = 6 years) after applying the exclusion criteria. Plasma 25(OH)D levels were measured in nmol/L which were applied before the mentioned conversion rate. Vitamin D insufficiency (<20 ng/L) was observed in 64% (42/65) of cancer patients and 63% (22/35) of controls at baseline. Notably, there was no noted difference between the synthesizing period (SP) and non-synthesizing period (NSP)—April 1st-September 30th and October 1st-March 31st, respectively. On the other hand, the control group had significant differences (median 26 SP vs. 57 NSP, *p* = 0.003) when comparing probes from different synthesis periods.

A case-control study by Mohan et al. [47] compared 25(OH)D levels in 102 children with cancer (aged 0–18 years, median = 9 years) to those in a control group of 51 healthy children. Vitamin D inadequacy was significantly more prevalent in the cancer group (80%) compared to the healthy controls (51%). A greater prevalence of vitamin D inadequacy was found in females (90%) than in males (74%).

A case report by Cook et al. [59] documented a 4-month-old African-American infant, exclusively breastfed, with a critically low 25(OH)D level of 6 ng/mL, showing significant improvement after oral supplementation and reaching a 97 ng/mL 25(OH)D level.

Helou et al. [49] in a 2014 cross-sectional study investigated vitamin D status in 89 pediatric cancer patients with a median age of 7 years. Vitamin D inadequacy (25(OH)D < 30 ng/mL) was prevalent in 72% of the study population, with 8% exhibiting severe deficiency (25(OH)D < 10 ng/mL). After adjusting for demographic and disease characteristics, logistic regression analysis revealed that children aged 6 years and older had 3.2 times higher odds of vitamin D deficiency.

### 3.4. Correlation Between Biological and Genetic Factors and Vitamin D Levels

Along with the previously mentioned risk factors of the studied populations, several very specific correlations were observed between 25(OH)D level subtypes of cancer along with vitamin D receptors’ polymorphism. These are listed in Table 6.

Solid tumors were consistently associated with higher inadequacy rates compared to hematologic malignancies [44,49].

Aristizabal et al. [44] found that among 115 patients receiving supplementation, those with solid tumors had levels 7 ng/mL lower than patients with hematologic malignancies (*p* = 0.003).

Similarly, Helou et al. [49] showed that solid tumors had a significant 61% vs. 39% reported inadequacy. A difference was noted for winter sampling, but it was not statistically significant.

In ALL patients, higher 25(OH)D levels were notably associated with favorable prognostic factors, such as B-cell lineage (*p* = 0.01) and hyperdiploidy (*p* = 0.02) [52,57,71,72,73]. On the other hand, T-cell ALL was often linked to a poorer prognosis, which correlated with significantly lower levels (*p* = 0.03) [56,73,74].

Malecka et al. [51] conducted a cross-sectional study assessing the vitamin D status in 59 children newly diagnosed with ALL. Vitamin D deficiency was identified in 39% of the patients. Children with higher 25(OH)D levels had a higher prevalence of B-cell phenotype (*p* = 0.01) and hyperdiploidy (*p* = 0.02). No significant seasonal influence was noted.

Bhattacharya et al. [56] analyzed 25(OH)D levels in 93 newly diagnosed ALL patients aged 0–15 years and examined their correlation with demographics, treatment, complications, and outcomes. At baseline, 95.5% of the patients had inadequate 25(OH)D levels, with a mean level of 14 ± 8 ng/mL. T-cell ALL was significantly associated with lower 25(OH)D levels, that is, 10 vs. 15 ng/mL (*p* = 0.03) when compared with B-cell ALL.

Genetic variations in the vitamin D receptor (VDR) affected the post-therapy vitamin D levels, with genotype-specific differences (e.g., the CT genotype showed a greater increase compared to the CC and TT genotypes) [53]. Supplementation improved the 25(OH)D levels, but the response varied by cancer type and VDR genotype.

Sherief et al.’s [53] study investigated the relationship between vitamin D receptor (VDR) genetic polymorphisms and osteonecrosis in 96 children with ALL. At diagnosis, 23% of the patients were vitamin D deficient (<20 ng/mL), and 50% had insufficient levels (<30 ng/mL). After consolidation therapy, their vitamin D sufficiency improved, with the proportion of patients achieving sufficient levels rising from 27% to 60%. Genotype-specific differences were observed: patients with the VDR CT genotype showed a mean increase of 10 ng/mL in 25(OH)D levels, those with the VDR CC genotype had a mean increase of 8 ng/mL, while the VDR TT genotype exhibited a mean decrease of 2 ng/mL.

### 3.5. Vitamin D and Clinical Outcomes

The included studies cover various cancer types along with clinical outcomes in relation to vitamin D. They include relapse risk, therapy duration, complications, and treatment-related adverse effects, and the findings are summarized in Table 7.

Across the studies, inadequate 25(OH)D levels in pediatric cancer patients are strongly associated with a poor prognosis, higher relapse risk (ALL, 1.2× higher, *p* = 0.04) [51], complications (51× higher likelihood) [40,45,50,51,52,56,58], and higher mortality rates (*p* = 0.02) [56].

A 2024 study by Nematollahi et al. [50] investigated the influence of 25(OH)D levels on the prognosis in 169 children newly diagnosed with ALL, compared to 189 healthy controls. The case group was followed for 36 months, with relapse-free survival as the primary outcome. While the mean 25(OH)D level in the ALL group (28 ± 19 ng/mL) was not significantly different from that of the control group (*p* = 0.7), the proportion of children with vitamin D inadequacy was significantly higher in the ALL group (46%) compared to that in the controls (26.5%). Among the ALL patients, 39% of those who relapsed had sufficient 25(OH)D levels, compared to 58% in the relapse-free group. Logistic regression analysis revealed that vitamin D deficiency was associated with a 1.2 times higher risk of relapse (*p* = 0.04). Further investigation is needed to clarify the association between vitamin D insufficiency and relapse risk, as the odds ratio for this group did not reach statistical significance, possibly due to the limited sample size.

Malecka et al. [51] found that ALL patients with optimal 25(OH)D levels experienced more severe thrombocytopenia (*p* = 0.02) and required platelet transfusions more often (*p* = 0.02).

Song et al. [52] conducted, in 2022, an observational cohort study which evaluated serum levels of multiple vitamins, including vitamin D, in 107 hospitalized children with mainly ALL (other diagnosis had no significant impact of the overall results). Of these, 52 children had an ongoing infection. Among the nine vitamins measured, only vitamin D showed a significant difference between the infected and non-infected groups, with a lower mean level (8 nmol/L lower) observed in the infected group (*p* = 0.03). Although lower 25(OH)D levels were observed in patients who did not achieve remission, this difference did not reach statistical significance (*p* = 0.07). No significant differences in 25(OH)D levels were found based on risk stratification or treatment regimen.

Several studies from our analysis suggest that lower 25(OH)D levels associate with more complications [40,56], longer treatment [47], a poorer prognosis [45], and higher mortality [56].

Oosterom et al. [40], in 2019, evaluated 25-hydroxyvitamin D and 24,25-dihydroxyvitamin D levels in 99 children with acute leukemia (mean age of 5.7 years) before and after methotrexate treatment. Patients who developed NCI grade 3 mucositis during therapy showed a significant mean decrease in 25(OH)D levels (−10 ± 14 nmol/L, *p* = 0.02). However, no overall significant difference in 25(OH)D levels was observed between patients with and without mucositis, suggesting that mucositis may increase the body’s vitamin D demand rather than directly reduce 25(OH)D levels.

Bhattacharya et al. [56] found that lower 25(OH)D levels were significantly associated with a higher mortality and complications during the induction phase (*p* = 0.02; *p* = 0.002) [57].

Jackman et al. [58] conducted a retrospective cohort study exploring the 25(OH)D levels in 295 leukemia patients and found significantly lower 25(OH)D levels in the AML group compared to the ALL group. For the AML, the mean level was 21 ng/mL, and 42% had inadequate levels (<20 ng/mL), compared to 25 ng/mL and 31% for the ALL group. The overall mean 25(OH)D levels across all patients was 24 ± 9 ng/mL, with 33% below 20 ng/mL.

In preschool-aged children, inadequate 25(OH)D levels were significantly associated with worse overall survival (OS) compared to normal levels (log-rank test, *p* = 0.03), as shown by Kaplan–Meier curves for all leukemia types and specific subtypes (ALL and AML).

Mohan et al. [47] compared the 25(OH)D levels between 102 children with cancer (ages 0–18, median of 9 years) and 51 healthy controls. Inadequate levels (<30 ng/mL) were associated with longer therapy durations (>1 year: 85% vs. <1 year: 64%).

Other findings by Juhász et al. 2020 [45] reveal through the retrospective analysis of 173 solid tumor patients, that inadequate 25(OH)D levels were 51 times more likely in patients with an unfavorable prognosis (progressive disease or death) than in those with favorable outcomes (complete or partial response, stable disease). Using linear regression, they also found that only 9.7% (*r*^2^ = 0.09) of the 25(OH)D variance can be attributed to PTH with a negative correlation.

## 4. Discussion

Vitamin D deficiency remains a significant public health concern, particularly in pediatric cancer patients, where intensive cancer therapies further deplete already compromised vitamin D reserves [75,76,77,78,79,80]. Our systematic review confirms a high prevalence of vitamin D deficiency in this population, consistent with previous research, and highlights its association with poorer immune function, suboptimal treatment response, and an increased risk of complications [49,81,82,83,84,85,86].

Vitamin D modulates inflammatory responses by influencing cytokine production. It reduces systemic inflammation by decreasing pro-inflammatory cytokines such as interleukin-12 (IL-12), tumor necrosis factor-alpha (TNF-α), and interleukin-6 (IL-6), while increasing anti-inflammatory cytokines like interleukin-10 (IL-10) [87,88]. This regulatory effect could explain the improvements in mucositis and bone density loss observed with vitamin D supplementation [40,89,90]. However, the variability in patient response complicates supplementation protocols, necessitating personalized approaches that consider baseline vitamin D levels, genetic receptor variations, and cancer type [91,92,93,94]. These findings call for a personalized approach to optimize immune function, improved treatment outcomes, and general well-being of pediatric patients [74,95,96].

Cancer and its treatments exert a heavy metabolic strain on the body, depleting nutritional reserves and impairing nutrient absorption due to side effects like appetite loss and organ dysfunction [75,76,77,78,79,94,97]. This highlights the critical need to address nutritional deficiencies, including vitamin D deficiency, in pediatric cancer patients. Notably, pediatric cancer patients receiving methotrexate or undergoing consolidation therapy with low 25(OH)D levels experience more frequent and severe complications, such as mucositis, which sometimes improve with supplementation [40,56].

Several factors influence the vitamin D status in pediatric cancer patients, including age, which is the most consistent finding, cancer type, and socioeconomic disparity. Similar to our findings, prior studies identified age and cancer type as significant determinants of vitamin D levels [98].

Other socioeconomic and demographic aspects also influence the vitamin D status. Children from lower-income households or non-Caucasian backgrounds exhibit a higher prevalence of deficiency, exhibiting the importance of equity-focused interventions and the importance of patient communication [42,44,58,83,99]. Even factors such as insurance type associate with 25(OH)D levels, with patients on public insurance exhibiting levels 4 ng/mL lower than those with private insurance (*p* = 0.04) [44].

Nutritional intake plays a pivotal role when considering prevention. Dietary intake for micronutrients such as calcium or vitamin D seems to differ significantly between patients with standard-risk and high-risk calls for a nutritional approach [100,101]. For younger children, rickets prophylaxis should be a key consideration [21,102,103,104,105,106,107], while older children often require increased vitamin D supplementation to counter the age-related decline in 25(OH)D levels [81].

Reduced outdoor activity is a documented contributor to vitamin D deficiency, as highlighted by a UK study reporting that only 27% of children engage in regular outdoor play [108,109]. Seasonal variations appear to have a minimal impact on 25(OH)D levels in pediatric cancer patients due to illness and treatment-related restrictions on UV light exposure [83,110,111,112,113]. Additionally, the role of UV light lamp treatment, such as that for managing mucositis, demands further research to better understand its impact on 25(OH)D levels.

This study highlights the varying degrees of vitamin D deficiency across different types of cancer, emphasizing the need for personalized screening and supplementation strategies. While it acknowledges the current lack of large-scale studies to definitively prove the effectiveness of vitamin D supplementation in cancer treatment, the available evidence suggests a link between solid tumors and an increased risk of vitamin D deficiency [114,115]. Given that adequate vitamin D levels are associated with improved outcomes in cancer patients, it is reasonable to consider that standardized supplementation protocols could lead to better treatment outcomes. However, careful monitoring and management of potential toxicity risks are crucial for patient safety. Essentially, the study suggests that a “one-size-fits-all” approach to vitamin D supplementation in cancer patients is not appropriate. Instead, personalized strategies based on individual needs and tumor types are necessary. Larger, more robust studies are needed to establish definitive guidelines for vitamin D supplementation in cancer care [116].

### 4.1. Strengths and Limitations

This review’s focus on diverse populations highlights health differences and enhances their relevance. It identifies research gaps, such as the effects of vitamin D on pediatric cancers and optimal supplementation strategies. Through the analysis of 20 diverse studies, this review underscores the high prevalence of vitamin D deficiency and its link to negative outcomes such as increased infections, reduced treatment effectiveness, and lower survival rates.

Our study has certain limitations. One of them is the inclusion of studies published in the last ten years, which may have excluded valuable older studies, but it was chosen to ensure the relevance and the most up-to-date findings in the context of the current clinical and research practice. Additionally, studies without available English translations were excluded, which introduced a regional bias. The study selection process, while systematic, is subject to human bias. The included studies differed in terms of study design, patient populations, and the vitamin D assessment method, which contribute to heterogeneity and potentially influence the generalizability of the findings. Lastly, the data on specific clinical outcomes, such as infection rate or survival, were limited, and this review focuses primarily on vitamin D levels, which without being directly linked to clinical outcomes like the quality of life or survival, may limit the real-world applicability.

### 4.2. Future Research Suggestions

Further large-scale randomized trials are ideal to validate these findings, but standardized supplementation protocols, common deficiency thresholds, and predetermined measurement time-points are the first steps in improving the quality of future studies.

Future research should assess the impact of vitamin D supplementation on pediatric cancer outcomes, including survival and quality of life, while tailoring interventions to individual factors like genetic variations and treatment stages. Studies should explore the possibility of preventive treatment and combined additional biomarkers, such as parathyroid hormone, vitamin D binding protein, C-reactive protein, interleukins, and so on, to deepen the understanding of vitamin D’s role. The mechanism of vitamin D deficiency in cancer development should be further investigated. Investigating geographical and ethnic variations in deficiency prevalence and long-term effects on survivors’ bone health, immunity, and quality of life will also help personalize the treatment.

## 5. Conclusions

This systematic review highlights the significant prevalence of vitamin D deficiency in pediatric cancer patients. Our analysis revealed that vitamin D deficiency is prevalent across various pediatric malignancies, with a consistent negative correlation between 25(OH)D levels and age. Addressing this deficiency through routine screening, personalized supplementation strategies, and further research has the potential to improve patient outcomes and enhance the overall quality of care in pediatric oncology.

Building on prior evidence [49], this study reported a notably high prevalence of hypovitaminosis D among pediatric oncology patients, making it safe to assume that this represents a risk population in need of a complex treatment approach.

Type of cancer, age, sex, race, and socioeconomic status are important indicators for identifying high-risk patients.

These findings underscore the importance of recognizing vitamin D deficiency as a significant clinical issue in pediatric oncology and implementing strategies to optimize the vitamin D status in this vulnerable population.

## Figures and Tables

**Table 1:** Thresholds for vitamin D status based on circulating levels of 25(OH)D.

Pediatric Endocrine Society [63]	Institute of Medicine [64]	Mayo Clinic [65]	The American Association of Clinical Endocrinologists [66]
Severe deficiency ≤5 ng/mL (≤12.5 nmol/L *) Deficiency ≤15 ng/mL (≤37.5 nmol/L *) Insufficiency 15–20 ng/mL (37.5–50.0 nmol/L *) Sufficiency 20–100 ng/mL (50–250 nmol/L *)	Deficiency <12 ng/mL (<30 nmol/L *) Insufficiency 12–20 ng/mL (30–50 nmol/L *) Sufficiency 20–50 ng/mL (50–125 nmol/L *)	Severe deficiency 10 ng/mL (25 nmol/L *) Deficiency 10–20 ng/mL (25–50 nmol/L *) Insufficiency 20–29 ng/mL (50–73 nmol/L *) Sufficiency 30–50 ng/mL (75–125 nmol/L *)	Deficiency ≤30 ng/mL (≤75 nmol/L *) Optimal Range ≥30 ng/mL (≥75 nmol/L *)

* Conversion factor: ng/mL = nmol/L × 0.401; nmol/L = ng/mL × 2.496.

**Table 2:** Summary of included studies. ALL = acute lymphoblastic leukemia; AML = acute myeloid leukemia; M = male; F = female; NA = not applicable; * marks use of median not mean; ** before exclusion for different reasons at the beginning and/or during the course of the study, *** remission group and nonremission group.

Study	Study Design	Age, Mean/Median *	Type of Cancer	Total Population **	Control Group	Study Group	Sex (%)	Mean 25(OH)D of the Study Group
Fullmer (2022) [42]	Cross-Sectional Retrospective	9 years	Multiple	544	408	136	M: 57%	22 ± 9 ng/mL
							F: 43%	
Bhandari (2021) [43]	Non-Randomized Control Trial	9 years	Multiple	169	136	29	M: 69%	28 ± 11 ng/mL
							F: 31%	
Aristizabal (2020) [44]	Cross-Sectional Retrospective	8 ± 5 years	Multiple	163	48	115	M: 55%	28 ± 12 ng/mL
							F: 45%	
Juhász (2020) [45]	Retrospective Observational Cohort	6 years	Solid Tumours	867	569	173	M: 56%	24 ± 11 ng/mL
							F: 45%	
Gurlek Gokcebay (2018) [46]	Prospective Observational Cohort	9 ± 5 years	Multiple	42	NA	42	M: 36%	14 ± 8 ng/mL
							F: 64%	
Mohan (2016) [47]	Case Control	9 years *	Multiple	102	51	51	M: 61%	80% Inadequacy
							F: 39%	
Iniesta (2016) [48]	Prospective Observational Cohort	4 years *	Multiple	117	33	65	M: 56%	64% Inadequacy 2
							F: 44%	
Helou (2014) [49]	Cross-Sectional	7 years *	Multiple	89	NA	89	M: 58%	43% Insufficient
							F: 42%	
Nematollahi (2024) [50]	Prospective Observational Cohort	58 ± 38 and 64 ± 38 (months)	ALL	358	189	169	M: 59%	28 ± 19 ng/mL
							F: 41%	
Malecka (2023) [51]	Cross-Sectional	6 ± 4 years	ALL	59	NA	59	M: NA	26 ± 12 ng/ml
							F: NA	
Song (2022) [52]	Observational Cohort	8 ± 9 and 9 ± 17 years	ALL	107	55	52	M: 57%	18 ± 8 and 15 ± 6 ng/mL ***
							F: 43%	
Sherief (2021) [53]	Cross-Sectional	8 ± 4 years	ALL	96	NA	96	M: 65%	23% Deficient
							F: 35%	
Maddheshiya (2021) [54]	Prospective Observational Cohort	6 years	ALL	80	30	50	M: 65%	32 ± 17 ng/mL
							F: 35%	
Norouzi (2021) [55]	Case Control	6 ± 4 years	ALL	60	30	30	M: 57%	20 ± 7 ng/mL
							F: 43%	
Bhattacharya (2020) [56]	Prospective Observational Cohort	6 ± 3 years	ALL	93	NA	93	M: 71%	14 ± 8 ng/mL
							F: 29%	
Oosterom (2019) [40]	Prospective Observational Cohort	6 years *	ALL	99	NA	99	M: 44%	25 ± 1 ng/mL
							F: 56%	
Orgel (2017) [57]	Randomized Control Study	15 years *	ALL	51	29	20	M: NA	20 ± 5 ng/mL
							F: NA	
Jackmann (2020) [58]	Cross-Sectional	7 ± 5 years	ALL&AML	295	NA	295	M: 58%	24 ± 9 ng/mL
							F: 42%	
Cook (2014) [59]	Case report	4 months	AML	1	NA	1	M: NA	6 ng/mL
Izurieta (2023) [60]	Retrospective Observational Cohort	5 years *	Neuroblastoma	182	NA	182	M: 60%	20 ng/mL *
							F: 40%	

**Table 3:** Vitamin D supplementation methods and effect in relation to initial levels.

Study	Year	Type of supplementation	Supplemented Patients (n)	Initial 25(OH)D Level (Mean ± SD)	Mean 25(OH)D Increase (ng/mL)	Measurement Time Interval
Bhandari [43]	2021	Oral Stoss Therapy (1 round adjusted high dose)	29	28 ± 11	+44	2 weeks
Aristizabal [44]	2020	Oral Weekly dose (3000–50,000 IU)	115	28 ± 12	+12	18 months
Juhász [45]	2020	Oral Daily dose (1000–2000 IU)	173	24	+4	4–6 weeks
Orgel [57]	2017	Intermittent Oral High Dose (10,000 IU/1 mL)	29	20 ± 5	+6 ± 2	≈7 months
Cook [59]	2014	Oral Daily Dose (2000 IU D3 2 weeks, 4000 IU D2 3 months)	1	6	+91	3.5 months

**Table 4:** Vitamin D supplementation regimens for pediatric patients undergoing hematopoietic stem cell transplantation. * If the total 25-OHD level was ≤30 ng/mL prior to day 100, the supplementation was initiated according to institutional guidelines.

Total Serum 25(OH)D	Single Oral Cholecalciferol Dose *	Mean Serum 25(OH)D	
		Initial	Achieved Pre-HSCT
<10 ng/mL	14,000 IU/kg/dose		
10–29 ng/mL	12,000 IU/kg/dose	28 ng/mL	72 ng/mL
30–50 ng/mL	7000 IU/kg/dose		

**Table 5:** Summary of studies on vitamin D levels and associated risk factors in pediatric oncology patients. * marks use of median not mean.

Study	Year	Population	Age, Mean/Median *	Type of Cancer	Mean ± SD 25(OH)D (ng/mL) /Deficiency/Insufficiency (%)	Findings
Izurieta [60]	2023	182	5 years *	Neuroblastoma	20*	Older children exhibited lower 25(OH)D levels (*p* = 0.004). No associations were found between 25(OH)D levels and race or sex.
Malecka [51]	2023	59	6 ± 4 years	ALL	26 ± 12	Older children exhibited lower 25(OH)D levels (*p* = 0.01). No seasonal differences in vitamin D status.
Fullmer [42]	2022	544	9 years	Multiple	22 ± 9	Older children exhibited lower 25(OH)D levels (*p* < 0.0001). Race correlation, particularly Black children, displayed the lowest levels (*p* < 0.005).
Aristizabal [44]	2021	163	8 ± 5 years	Multiple	28 ± 12	Older age (>10 years, *p* = 0.019), Hispanic ethnicity (*p* = 0.002), and female sex (*p* = 0.005) were associated with lower levels. Public insurance correlated with lower levels (*p* = 0.04).
Maddheshiya [54]	2021	80	6 years	ALL	32 ± 17	Female sex was identified as a risk factor for vitamin D deficiency.
Norouzi [55]	2021	60	6 ± 4 years	ALL	20 ± 7	Age older than 10 is a significant risk factor.
Bhattacharya [56]	2020	93	6 ± 3 years	ALL	14 ± 8	Correlation between age and 25(OH)D levels. Older kids have lower levels of 25(OH)D (*p* = 0.007). Female gender was a risk factor for inadequacy (*p* = 0.036).
Jackmann [58]	2020	295	7 ± 5 years	ALL& AML	24 ± 9	Older age was also associated with lower 25(OH)D levels, with a prevalence of deficiency of 23% in preschool children versus 45% in school-aged children (*p* < 0.001). Season (winter compared to summer) and recent calendar years are predictors for a lower 25(OH)D level (*p* = 0.001).
Juhász [45]	2020	867	6 years	Solid Tumors	24 ± 11	Seasonal changes do not correlate with a lower 25(OH)D.
Oosterom [40]	2019	99	6 years *	ALL	25 ± 1	More frequent inadequacy at > 4 years of age as compared to children aged between 1 and 4 years (*p* < 0.001).
Gurlek [46]	2018	42	9 ± 5 years	Multiple	14 ± 8	No significant differences based on sex, age, season, or urban/rural residence.
Iniesta [48]	2016	117	4 years *	Multiple	64% Insufficiency SG 63% Insufficiency CG	Older children exhibited lower 25(OH)D levels (*p* < 0.001). Seasonal variation in 25(OH)D levels was significant in controls (median 57 nmol/L during synthesizing vs. 26 nmol/L during non-synthesizing periods (*p* = 0.003).
Mohan [47]	2016	102	9 years *	Multiple	80% Inadequacy	Greater prevalence of vitamin D inadequacy in females (90%) than in males (74%).
Cook [59]	2014	1	4 months	AML	6	Black race association with lower 25(OH)D levels.
Helou [49]	2014	89	7 years *	Multiple	43% insufficiency	Hypovitaminosis associated with age (≥6 years, 3.2× higher odds) and Black children.

**Table 6:** Vitamin D levels and cancer-specific characteristics.

Study	Year	Cancer Type	Mean ± SD 25(OH)D (ng/mL) /Deficiency (%)	Correlation
Malecka [51]	2023	ALL	39% Deficient	Higher levels associated with a B-cell phenotype and hyperdiploidy.
Aristizabal [44]	2021	Multiple	28 ± 12	Solid tumors had 7 ng/mL lower 25(OH)D levels than hematologic malignancies with little seasonal variation observed (*p* = 0.003).
Sherief [53]	2021	ALL	23% deficient	VDR polymorphisms influenced post-therapy vitamin D changes.
Bhattacharya [56]	2020	ALL	14 ± 8	T-cell ALL (*p* = 0.027) associated with lower 25(OH)D.
Helou [49]	2014	ALL and AML	24 ± 9	Solid tumors showed higher rates of inadequacy (61% vs. 39%).

**Table 7:** Type of cancer and notable clinical outcomes.

Study	Year	Cancer Type	Clinical Outcome Correlation
Nematollahi [50]	2024	ALL	Deficiency associated with a 1.2 times higher risk of relapse; sufficiency correlated with better outcomes.
Malecka [51]	2023	ALL	Optimal 25(OH)D concentrations experienced more severe thrombocytopenia (*p* = 0.02) and required platelet transfusions more frequently (*p* = 0.02)
Song [52]	2022	ALL	Lower 25(OH)D levels were significantly associated with infections (*p* = 0.03).
Bhattacharya [56]	2020	ALL	Vitamin D inadequacy (<30 ng/mL) was linked to a higher mortality during the induction phase and complications (*p* = 0.002)
Jackmann [58]	2020	ALL& AML	In preschool-aged children, inadequate vitamin D levels were significantly linked to a worse overall survival (OS) across all the leukemia types, including ALL and AML (log-rank test, *p* = 0.03)
Juhász [45]	2020	Solid Tumors	Patients with inadequacy (<30 ng/mL) were 51 times more likely to have a poor prognosis.
Oosterom [40]	2019	ALL	A decrease in 25(OH)D levels was significantly associated with severe oral mucositis (*p* = 0.012).
Mohan [47]	2016	Multiple	Prolonged therapy duration (>1 year) was linked to significantly lower levels.

## Data Availability

The original contributions presented in the study are included in the article. Further inquiries can be directed to the corresponding author.

## Supplementary Materials

The following supporting information can be downloaded at https://www.mdpi.com/article/10.3390/cancers16244201/s1, Table S1: Quality Assessment of Included Studies. Y = yes; N = no; CD = cannot determine; NA = not applicable; NR = not reported.

## References

[1] Bouillon R., Marcocci C., Carmeliet G., Bikle D., White J.H., Dawson-Hughes B., Lips P., Munns C.F., Lazaretti-Castro M., Giustina A. (2019). Skeletal and Extraskeletal Actions of Vitamin D: Current Evidence and Outstanding Questions. Endocr. Rev..

[2] Khazai N., Judd S.E., Tangpricha V. (2008). Calcium and vitamin D: Skeletal and extraskeletal health. Curr. Rheumatol. Rep..

[3] Wei F., Wang Z., Wang J., Xu H., Zhou H. (2018). Serum vitamin D levels among children aged 0–12 years in the First Affiliated Hospital of Harbin Medical University, China. J. Public Health.

[4] Kulda V. (2012). Vitamin D metabolism. Vnitr. Lek..

[5] Muresan G., Hedesiu M., Lucaciu O., Boca S., Petrescu N. (2022). Effect of Vitamin D on Bone Regeneration: A Review. Medicina.

[6] Townsend K., Evans K.N., Campbell M.J., Colston K.W., Adams J.S., Hewison M. (2005). Biological actions of extra-renal 25-hydroxyvitamin D-1α-hydroxylase and implications for chemoprevention and treatment. J. Steroid Biochem. Mol. Biol..

[7] Sassi F., Tamone C., D’Amelio P. (2018). Vitamin D: Nutrients, Hormone, and Immunomodulator. Nutrients.

[8] Bikle D.D. (2022). Vitamin D Regulation of Immune Function. Curr. Osteoporos. Rep..

[9] Jeffery L.E., Raza K. (2016). Hewison Vitamin D in rheumatoid arthritis—towards clinical application. Nat. Rev. Rheumatol..

[10] Chen J., Tang Z., Slominski A.T., Li W., Żmijewski M.A., Liu Y., Chen J. (2020). Vitamin D and its analogs as anticancer and anti-inflammatory agents. Eur. J. Med. Chem..

[11] Bortman P., Folgueira M.A.A.K., Katayama M.L.H., Snitcovsky I.M.L., Brentani M.M. (2002). Antiproliferative effects of 1,25-dihydroxyvitamin D3 on breast cells: A mini review. Braz. J. Med. Biol. Res..

[12] Sundar R., Rai A.B., Kumar J.N., Divakar D.D. (2023). The role of Vitamin D as an adjunct for bone regeneration: A systematic review of literature. Saudi Dent. J..

[13] Delrue C., Speeckaert M.M. (2023). Vitamin D and Vitamin D-Binding Protein in Health and Disease. Int. J. Mol. Sci..

[14] Zerwekh J.E. (2008). Blood biomarkers of vitamin D status. Am. J. Clin. Nutr..

[15] Martineau A.R., Wilkinson R.J., Wilkinson K.A., Newton S.M., Kampmann B., Hall B.M., Packe G.E., Davidson R.N., Eldridge S.M., Maunsell Z.J. (2007). A Single Dose of Vitamin D Enhances Immunity to Mycobacteria. Am. J. Respir. Crit. Care Med..

[16] Raimondi S., Johansson H., Maisonneuve P., Gandini S. (2009). Review and meta-analysis on vitamin D receptor polymorphisms and cancer risk. Carcinogenesis.

[17] Serrano D., Gnagnarella P., Raimondi S., Gandini S. (2016). Meta-analysis on vitamin D receptor and cancer risk. Eur. J. Cancer Prev..

[18] Feldman D., Krishnan A.V., Swami S., Giovannucci E., Feldman B.J. (2014). The role of vitamin D in reducing cancer risk and progression. Nat. Rev. Cancer.

[19] Haussler M.R., Jurutka P.W., Mizwicki M., Norman A.W. (2011). Vitamin D receptor (VDR)-mediated actions of 1α,25(OH)_2_vitamin D3: Genomic and non-genomic mechanisms. Best Pract. Res. Clin. Endocrinol. Metab..

[20] Xia X., Xu F., Dai D., Xiong A., Sun R., Ling Y., Qiu L., Wang R., Ding Y., Lin M. (2024). VDR is a potential prognostic biomarker and positively correlated with immune infiltration: A comprehensive pan-cancer analysis with experimental verification. Biosci. Rep..

[21] Marazziti D., Mangiapane P., Carbone M.G., Morana F., Arone A., Massa L., Palermo S., Violi M., Bertini G., Massoni L. (2021). Vitamin D: A Pleiotropic Hormone with Possible Psychotropic Activities. Curr. Med. Chem..

[22] Liu W., Chen Y., Golan M.A., Annunziata M.L., Du J., Dougherty U., Kong J., Much M., Huang Y., Pekow J. (2013). Intestinal epithelial vitamin D receptor signaling inhibits experimental colitis. J. Clin. Investig..

[23] Fleet J.C., Desmet M., Johnson R., Li Y. (2012). Vitamin D and cancer: A review of molecular mechanisms. Biochem. J..

[24] Young M.R.I., Xiong Y. (2018). Influence of vitamin D on cancer risk and treatment: Why the variability?. Trends Cancer Res..

[25] Seyedalipour F., Mansouri A., Vaezi M., Gholami K., Heidari K., Hadjibabaie M., Ghavamzadeh A. (2017). High Prevalence of Vitamin D Deficiency in Newly Diagnosed Acute Myeloid Leukemia Patients and Its Adverse Outcome. Int. J. Hematol. Oncol. Stem Cell Res..

[26] Aliashrafi S., Ebrahimi-Mameghani M. (2017). 7: A systematic review on vitamin D and angiogenesis. BMJ Open.

[27] Marigoudar J.B., Sarkar D., Yuguda Y.M., Abutayeh R.F., Kaur A., Pati A., Mitra D., Ghosh A., Banerjee D., Borah S. (2022). Role of vitamin D in targeting cancer and cancer stem cell populations and its therapeutic implications. Med. Oncol..

[28] Garland C.F., Garland F.C., Gorham E.D., Lipkin M., Newmark H., Mohr S.B., Holick M.F. (2006). The Role of Vitamin D in Cancer Prevention. Am. J. Public Health.

[29] Schwartz G.G., Whitlatch L.W., Chen T.C., Lokeshwar B.L., Holick M.F. (1998). Human prostate cells synthesize 1,25-dihydroxyvitamin D3 from 25-hydroxyvitamin D3. Cancer Epidemiol. Biomark. Prev..

[30] Hsu J.Y., Feldman D., McNeal J.E., Peehl D.M. (2001). Reduced 1alpha-hydroxylase activity in human prostate cancer cells correlates with decreased susceptibility to 25-hydroxyvitamin D3-induced growth inhibition. Cancer Res..

[31] Ogunkolade B., Boucher B.J., Fairclough P.D., Hitman G.A., Dorudi S., Jenkins P.J., Bustin S.A. (2002). Expression of 25-hydroxyvitamin D-1-α-hydroxylase mRNA in individuals with colorectal cancer. Lancet.

[32] Bareis P., Bises G., Bischof M.G., Cross H.S., Peterlik M. (2001). 25-Hydroxy-Vitamin D Metabolism in Human Colon Cancer Cells during Tumor Progression. Biochem. Biophys. Res. Commun..

[33] Tangpricha V., Flanagan J.N., Whitlatch L.W., Tseng C.C., Chen T.C., Holt P.R., Lipkin M.S., Holick M.F. (2001). 25-hydroxyvitamin D-1α-hydroxylase in normal and malignant colon tissue. Lancet.

[34] Bajbouj K., Al-Ali A., Shafarin J., Sahnoon L., Sawan A., Shehada A., Elkhalifa W., Saber-Ayad M., Muhammad J.S., Elmoselhi A.B. (2022). Vitamin D Exerts Significant Antitumor Effects by Suppressing Vasculogenic Mimicry in Breast Cancer Cells. Front. Oncol..

[35] Muñoz A., Grant W.B. (2022). Vitamin D and Cancer: An Historical Overview of the Epidemiology and Mechanisms. Nutrients.

[36] Carlberg C., Muñoz A. (2022). An update on vitamin D signaling and cancer. Semin. Cancer Biol..

[37] Hansson M.E.A., Norlin A.-C., Omazic B., Wikström A.-C., Bergman P., Winiarski J., Remberger M., Sundin M. (2014). Vitamin D Levels Affect Outcome in Pediatric Hematopoietic Stem Cell Transplantation. Biol. Blood Marrow Transplant..

[38] Duncan C.N., Vrooman L., Apfelbaum E.M., Whitley K., Bechard L., Lehmann L.E. (2011). 25-Hydroxy Vitamin D Deficiency Following Pediatric Hematopoietic Stem Cell Transplant. Biol. Blood Marrow Transplant..

[39] Hamidieh A.A., Sherafatmand M., Mansouri A., Hadjibabaie M., Ashouri A., Jahangard-Rafsanjani Z., Gholami K., Javadi M.R., Ghavamzadeh A., Radfar M. (2016). Calcitriol for Oral Mucositis Prevention in Patients With Fanconi Anemia Undergoing Hematopoietic SCT: A Double-Blind, Randomized, Placebo-Controlled Trial. Am. J. Ther..

[40] Oosterom N., Lee A.M.C., Xu X., Hua B., Tapp H., Wen X.S., Xian C.J. (2019). A decrease in vitamin D levels is associated with methotrexate-induced oral mucositis in children with acute lymphoblastic leukemia. Support. Care Cancer.

[41] van Atteveld J.E., Verhagen I.E., van den Heuvel-Eibrink M.M., van Santen H.M., van der Sluis I.M., Di Iorgi N., Simmons J.H., Ward L.M., Neggers S.J.C.M.M. (2021). Vitamin D supplementation for children with cancer: A systematic review and consensus recommendations. Cancer Med..

[42] Fullmer M., Su A., Bachrach S., Hossain J., Kecskemethy H.H. (2022). Newly Diagnosed Children with Cancer Have Lower 25-Vitamin D Levels than Their Cancer-Free Peers: A Comparison across Age, Race, and Sex. Cancers.

[43] Bhandari R., Aguayo-Hiraldo P., Malvar J., Cheng K., Sacapano A., Abdel-Azim H., Chi Y.Y., Wallace G., Asgharzadeh S., Jodele S. (2021). Ultra-High Dose Vitamin D in Pediatric Hematopoietic Stem Cell Transplantation: A Nonrandomized Controlled Trial. Transpl. Cell Ther..

[44] Aristizabal P., Sherer M., Perdomo B.P., Castelao E., Thornburg C.D., Proudfoot J., Jacobs E., Newfield R.S., Zage P., Roberts W. (2020). Sociodemographic and clinical characteristics associated with vitamin D status in newly diagnosed pediatric cancer patients. Pediatr. Hematol. Oncol..

[45] Juhász O., Jakab Z., Szabó A., Garami M. (2020). Examining the Vitamin D Status of Children With Solid Tumors. J. Am. Coll. Nutr..

[46] Gokcebay D.G., Emir S., Bayhan T., Demir H.A., Ozyoruk D., Gunduz M., Koc N. (2018). Evaluation of Serum Trace Element and Vitamin Levels in Children With Cancer in the First 6 Months After Diagnosis. J. Pediatr. Hematol. Oncol..

[47] Mohan R., Mohan G., Scott J., Rajendran A., Paramasivam V., Ravindran M. (2016). Vitamin D insufficiency among children with cancer in India. Indian J. Med. Paediatr. Oncol..

[48] Iniesta R.R., Paciarotti I., Davidson I., McKenzie J.M., Brand C., Chin R.F., Brougham M.F., Wilson D.C. (2016). 5-Hydroxyvitamin D concentration in paediatric cancer patients from Scotland: A prospective cohort study. Br. J. Nutr..

[49] Helou M., Ning Y., Yang S., Irvine P., Bachmann L.M., Godder K., Massey G. (2014). Vitamin D Deficiency in Children With Cancer. J. Pediatr. Hematol. Oncol..

[50] Nematollahi P., Arabi S., Mansourian M., Yousefian S., Moafi A., Mostafavi S.N., Alavi Naeini A., Ebrahimi A., Ebrahimpour K., Amin M.M. (2024). Potential role of serum vitamin D as a risk factor in pediatric acute lymphoblastic leukemia. Pediatr. Hematol. Oncol..

[51] Malecka A., Hennig M., Jaworski R., Irga-Jaworska N. (2023). The Vitamin D Status in Children With Newly Diagnosed Acute Lymphoblastic Leukemia and Its Potential Impact on the Primary Symptoms of Leukemia and Course of Induction Treatment. J. Pediatr. Hematol. Oncol..

[52] Song Z., Li J., Cao J., Zhang L., Zhang Z., Feng S., Zhong D., Yue M., Hu M., Liu R. (2022). Analysis of Multiple Vitamins Serum Levels and Disease-Related Factors in Children with Acute Leukemia. J. Healthc. Eng..

[53] Sherief L.M., Beshir M., Raafat N., Abdelkhalek E.R., Mokhtar W.A., Elgerby K.M., Soliman B.K., Salah H.E., Mokhtar G.A., Kamal N.M. (2021). Genetic polymorphism of vitamin D receptors and plasminogen activator inhibitor-1 and osteonecrosis risk in childhood acute lymphoblastic leukemia. Mol. Genet. Genom. Med..

[54] Maddheshiya S., Singh S.K., Kumar I., Aggarwal P., Gupta V. (2021). Bone Mineral Metabolism During Chemotherapy in Childhood Acute Lymphoblastic Leukemia. J. Pediatr. Hematol. Oncol..

[55] Norouzi A., Motaghi M., Hassanshahi G., Nazari-Robati M. (2021). Exploring the expression profile of vitamin D receptor and its related long non-coding RNAs in patients with acute lymphoblastic leukemia. Rev. Assoc. Med. Bras..

[56] Bhattacharya S., Verma N., Kumar A. (2020). Prevalence of vitamin D deficiency in childhood acute lymphoblastic leukemia and its association with adverse outcomes during induction phase of treatment. Nutr. Cancer.

[57] Orgel E., Mueske N.M., Sposto R., Gilsanz V., Wren T.A.L., Freyer D.R., Butturini A.M., Mittelman S.D. (2017). A randomized controlled trial testing an adherence-optimized Vitamin D regimen to mitigate bone change in adolescents being treated for acute lymphoblastic leukemia. Leuk. Lymphoma.

[58] Jackmann N., Mäkitie O., Harila-Saari A., Gustafsson J., Dernroth D.N., Frisk P. (2020). Vitamin D status in children with leukemia, its predictors, and association with outcome. Pediatr. Blood Cancer.

[59] Cook C., Bernardo V., Shelso J., Ribeiro R.C. (2014). Vitamin D Deficiency and Hypocalcemia in an Infant With Newly Diagnosed AML. J. Pediatr. Hematol. Oncol..

[60] Izurieta-Pacheco A.C., Sangrós-Gimenez A., Martínez-Garcia E., Perez-Jaume S., Mora J., Gorostegui-Obanos M. (2023). Vitamin D Status in Children With High-risk Neuroblastoma. J. Pediatr. Hematol. Oncol..

[61] Gospodarska E., Dastidar R.G., Carlberg C. (2023). Intervention Approaches in Studying the Response to Vitamin D3 Supplementation. Nutrients.

[62] Manson J.E., Cook N.R., Lee I.M., Christen W., Bassuk S.S., Mora S., Gibson H., Gordon D., Copeland T., D‘Agostino D. (2019). Vitamin D Supplements and Prevention of Cancer and Cardiovascular Disease. N. Engl. J. Med..

[63] Misra M., Pacaud D., Petryk A., Collett-Solberg P.F., Kappy M. (2008). Vitamin D Deficiency in Children and Its Management: Review of Current Knowledge and Recommendations. Pediatrics.

[64] Ross A.C., Taylor C.L., Yaktine A.L., Del Valle H.B., Institute of Medicine (US) Committee to Review Dietary Reference Intakes for Vitamin D and Calcium (2011). Dietary Reference Intakes for Calcium and Vitamin D.

[65] Kennel K.A., Drake M.T., Hurley D.L. (2010). Vitamin D Deficiency in Adults: When to Test and How to Treat. Mayo Clin. Proc..

[66] Camacho P.M., Petak S.M., Binkley N., Diab D.L., Eldeiry L.S., Farooki A., Harris S.T., Hurley D.L., Kelly J., Lewiecki E.M. (2020). American Association of Clinical Endocrinologists/American College of Endocrinology Clinical Practice Guidelines for the Diagnosis and Treatment of Postmenopausal Osteoporosis—2020 Update. Endocr. Pract..

[67] Page M.J., McKenzie J.E., Bossuyt P.M., Boutron I., Hoffmann T.C., Mulrow C.D., Shamseer L., Tetzlaff J.M., Akl E.A., Brennan S.E. (2021). The PRISMA 2020 statement: An updated guideline for reporting systematic reviews. BMJ.

[68] Cole T.J. (2015). Too many digits: The presentation of numerical data. Arch. Dis. Child..

[69] Wallace G., Jodele S., Myers K.C., Dandoy C.E., El-Bietar J., Nelson A., Teusink-Cross A., Khandelwal P., Taggart C., Gordon C.M. (2018). Single Ultra-High-Dose Cholecalciferol to Prevent Vitamin D Deficiency in Pediatric Hematopoietic Stem Cell Transplantation. Biol. Blood Marrow Transplant..

[70] Macris P.C., McMillen K. (2012). Hematopoietic Stem Cell Transplantation Nutrition Care Criteria.

[71] Dores G.M., Devesa S.S., Curtis R.E., Linet M.S., Morton L.M. (2012). Acute leukemia incidence and patient survival among children and adults in the United States, 2001–2007. Blood.

[72] Dastugue N., Suciu S., Plat G., Speleman F., Cavé H., Girard S., Bakkus M., Pagès M.P., Yakouben K., Nelken B. (2013). Hyperdiploidy with 58-66 chromosomes in childhood B-acute lymphoblastic leukemia is highly curable: 58951 CLG-EORTC results. Blood.

[73] Raetz E.A., Teachey D.T. (2016). T-cell acute lymphoblastic leukemia. Hematol. Am. Soc. Hematol. Educ. Program.

[74] Jinca C., Petrescu C.A.M., Boeriu E., Oprisoni A., Balint-Gib L., Baica M., Popa C., Andreescu N., Serban M., Ursu E. (2018). The impact of immunological and biomolecular investigations on the outcome of children with acute lymphoblastic leukemia—experience of IIIrd Paediatric Clinic Timisoara. Romanian Rev. Lab. Med..

[75] Goldsby R., Chen Y., Raber S., Li L., Diefenbach K., Shnorhavorian M., Kadan-Lottick N., Kastrinos F., Yasui Y., Stovall M. (2011). Survivors of Childhood Cancer Have Increased Risk of Gastrointestinal Complications Later in Life. Gastroenterology.

[76] Pedretti L., Massa S., Leardini D., Muratore E., Rahman S., Pession A., Esposito S., Masetti R. (2023). Role of Nutrition in Pediatric Patients with Cancer. Nutrients.

[77] Triarico S., Rinninella E., Attinà G., Romano A., Maurizi P., Mastrangelo S., Ruggiero A. (2022). Nutritional status in the pediatric oncology patients. Front. Biosci..

[78] Rogers P.C., Barr R.D. (2020). The relevance of nutrition to pediatric oncology: A cancer control perspective. Pediatr. Blood Cancer.

[79] Kandemir I., Anak S., Karaman S., Yaman A., Varkal M.A., Devecioglu O. (2023). Nutritional Status of Pediatric Patients With Acute Lymphoblastic Leukemia Under Chemotherapy: A Pilot Longitudinal Study. J. Pediatr. Hematol. Oncol..

[80] Delvin E., Alos N., Rauch F., Marcil V., Morel S., Boisvert M., Lecours M.A., Laverdière C., Sinnett D., Krajinovic M. (2019). Vitamin D nutritional status and bone turnover markers in childhood acute lymphoblastic leukemia survivors: A PETALE study. Clin. Nutr..

[81] Alfredsson L., Armstrong B.K., Butterfield D.A., Chowdhury R., de Gruijl F.R., Feelisch M., Garland C.F., Hart P.H., Hoel D.G., Jacobsen R. (2020). Insufficient Sun Exposure Has Become a Real Public Health Problem. Int. J. Environ. Res. Public Health.

[82] Choi H.S., Oh H.J., Choi H., Choi W.H., Kim J.G., Kim K.M., Kim K.J., Rhee Y., Lim S.K. (2011). Vitamin D Insufficiency in Korea—A Greater Threat to Younger Generation: The Korea National Health and Nutrition Examination Survey (KNHANES) 2008. J. Clin. Endocrinol. Metab..

[83] Subramanian A., Burrowes H.B., Rumph J.T., Wilkerson J., Jackson C.L., Jukic A.M.Z. (2024). Vitamin D Levels in the United States: Temporal Trends (2011–2018) and Contemporary Associations with Sociodemographic Characteristics (2017–2018). Nutrients.

[84] Looned K., Banerjee A., Landge J., Pandit D. (2017). Intergenerational Decline in Vitamin D Status: A Cross-Sectional Study Among Medical Students and Their Teachers. Int. J. Nutr. Pharmacol. Neurol. Dis..

[85] Arshad S., Zaidi S.J.A. (2022). Vitamin D levels among children, adolescents, adults, and elders in Pakistani population: A cross-sectional study. BMC Public Health.

[86] Kárász N., Juhász O., Imrei M., Garami M. (2023). Long-Term Prognosis in Relation to Vitamin D Status in Pediatric Solid Tumor Patients. Nutrients.

[87] Querfeld U. (2013). Vitamin D and inflammation. Pediatr. Nephrol..

[88] Vojinovic J., Cimaz R. (2015). Vitamin D—Update for the pediatric rheumatologists. Pediatr. Rheumatol..

[89] Kelly P.M., Pottenger E. (2022). Bone Health Issues in the Pediatric Oncology Patient. Semin. Oncol. Nurs..

[90] Marcucci G., Beltrami G., Tamburini A., Body J.J., Confavreux C.B., Hadji P., Holzer G., Kendler D., Napoli N., Pierroz D.D. (2019). Bone health in childhood cancer: Review of the literature and recommendations for the management of bone health in childhood cancer survivors. Ann. Oncol..

[91] Zgaga L. (2020). Heterogeneity of the Effect of Vitamin D Supplementation in Randomized Controlled Trials on Cancer Prevention. JAMA Netw. Open.

[92] Vaughan-Shaw P.G., O‘Sullivan F., Farrington S.M., Theodoratou E., Campbell H., Dunlop M.G., Zgaga L. (2017). The impact of vitamin D pathway genetic variation and circulating 25-hydroxyvitamin D on cancer outcome: Systematic review and meta-analysis. Br. J. Cancer.

[93] Voltan G., Cannito M., Ferrarese M., Ceccato F., Camozzi V. (2023). Vitamin D: An Overview of Gene Regulation, Ranging from Metabolism to Genomic Effects. Genes.

[94] Tah P.C., Shanita S.N., Poh B.K. (2012). Nutritional status among pediatric cancer patients: A comparison between hematological malignancies and solid tumors. J. Spéc. Pediatr. Nurs..

[95] Kourti M., Hatzipantelis E. (2023). Evolving Aspects of Prognostic Factors for Pediatric Cancer. Diagnostics.

[96] Toma A.-O., Boeriu E., Decean L., Bloanca V., Bratosin F., Levai M.C., Vasamsetti N.G., Alambaram S., Oprisoni A.L., Miutescu B. (2023). The Effects of Lack of Awareness in Age-Related Quality of Life, Coping with Stress, and Depression among Patients with Malignant Melanoma. Curr. Oncol..

[97] Brinksma A., Petrie F., Roodbol E., Sulkers E., Kamps W.A., de Bont E.S.J.M., Boot A.M., Burgerhof J.G.M., Tamminga R.Y.J., Tissing W.J.E. (2015). Changes in nutritional status in childhood cancer patients: A prospective cohort study. Clin. Nutr..

[98] Iniesta R.R., Rush R., Paciarotti I., Rhatigan E.B., Brougham F.H.M., McKenzie J.M., Wilson D.C. (2016). Systematic review and meta-analysis: Prevalence and possible causes of vitamin D deficiency and insufficiency in pediatric cancer patients. Clin. Nutr..

[99] Boeriu E., Borda A., Miclea E., Boeriu A.I., Vulcanescu D.D., Bagiu I.C., Horhat F.G., Kovacs A.F., Avram C.R., Diaconu M.M. (2023). Prognosis Communication in Pediatric Oncology: A Systematic Review. Children.

[100] Saternus R., Vogt T., Reichrath J. (2019). A Critical Appraisal of Strategies to Optimize Vitamin D Status in Germany, a Population with a Western Diet. Nutrients.

[101] Ladas E.J., Orjuela M., Stevenson K., Cole P.D., Lin M., Athale U.H., Clavell L.A., Leclerc J.M., Michon B., Schorin M.A. (2016). Dietary intake and childhood leukemia: The Diet and Acute Lymphoblastic Leukemia Treatment (DALLT) cohort study. Nutrition.

[102] De Rooij J., Zwaan C., Van den Heuvel-Eibrink M. (2015). Pediatric AML: From Biology to Clinical Management. J. Clin. Med..

[103] National Cancer Institute (2002). Childhood Acute Myeloid Leukemia Treatment (PDQ^®^)—Health Professional Version.

[104] Pettifor J.M. (2013). Nutritional rickets: Pathogenesis and prevention. Pediatr. Endocrinol. Rev..

[105] Munns C.F., Shaw N., Kiely M., Specker B.L., Thacher T.D., Ozono K., Michigami T., Tiosano D., Mughal M.Z., Mäkitie O. (2016). Global Consensus Recommendations on Prevention and Management of Nutritional Rickets. J. Clin. Endocrinol. Metab..

[106] Gupta P., Dabas A., Seth A., Bhatia V.L., Khadgawat R., Kumar P., Balasubramanian S., Khadilkar V., Mallikarjuna H.B., Godbole T. (2022). Indian Academy of Pediatrics Revised (2021) Guidelines on Prevention and Treatment of Vitamin D Deficiency and Rickets. Indian Pediatr..

[107] Thacher T.D., Fischer P.R., Pettifor J.M., Lawson J.O., Isichei C.O., Chan G.M. (2000). Case-control study of factors associated with nutritional rickets in Nigerian children. J. Pediatr..

[108] Tremblay M., Gray C., Babcock S., Barnes J., Bradstreet C.C., Carr D., Chabot G., Choquette L., Chorney D., Collyer C. (2015). Position Statement on Active Outdoor Play. Int. J. Environ. Res. Public Health.

[109] Dodd H.F., FitzGibbon L., Watson B.E., Nesbit R.J. (2021). Children’s Play and Independent Mobility in 2020: Results from the British Children’s Play Survey. Int. J. Environ. Res. Public Health.

[110] Young A.R., Narbutt J., Harrison G.I., Lawrence K.P., Bell M., O‘Connor C., Olsen P., Grys K., Baczynska K.A., Rogowski-Tylman M. (2019). Optimal sunscreen use, during a sun holiday with a very high ultraviolet index, allows vitamin D synthesis without sunburn. Br. J. Dermatol..

[111] Hogan A. The Trending Sunscreen Brands of Summer 2024 According to Data. NewBeauty. https://www.newbeauty.com/trending-sunscreens-2024/.

[112] Johnson E. The 13 Best Sunscreens of 2024, Tested in Hawaii. PEOPLE. https://people.com/best-sunscreens-8636209.

[113] de Gatta C.A.F., de Mena H.E., Calderón O.G., Díaz J.A., Hernández-Fabián A., Peral R.T. (2023). Vitamina D en pediatría tras el fin del confinamiento domiciliario: Estudio prospectivo. Andes Pediatr..

[114] Olsen B., Bodea J., Garcia A., Beebe K., Campbell C., Schwalbach C., Salzberg D., Miller H., Adams R., Mirea L. (2022). Vitamin D Supplementation: Association With Serum Cytokines in Pediatric Hematopoietic Stem Cell Transplantation. Front. Pediatr..

[115] Shliakhtsitsava K., Fisher E.S., Trovillion E.M., Bush K., Kuo D.J., Newfield R.S., Thornburg C.D., Roberts W., Aristizabal P. (2021). Improving vitamin D testing and supplementation in children with newly diagnosed cancer: A quality improvement initiative at Rady Children’s Hospital San Diego. Pediatr. Blood Cancer.

[116] Vogiatzi M.G., Jacobson-Dickman E., DeBoer M.D. (2014). Vitamin D Supplementation and Risk of Toxicity in Pediatrics: A Review of Current Literature. J. Clin. Endocrinol. Metab..

