# Supplementary material for: Are Pediatric Cancer Patients a Risk Group for Vitamin D Deficiency? A Systematic Review
Source: Cancers (Basel). 2024 Dec 17;16(24):4201. doi: 10.3390/cancers16244201 (PMC11674856; doi:10.3390/cancers16244201)
Supplement: Supplementary file 1 [file cancers-16-04201-s001.zip › cancers-3321969-supplementary.pdf]

Systematic Review

Are Pediatric Cancer Patients a Risk Group for Vitamin D Deficiency? A Systematic Review

Supplemental Table S1. Quality Assessment of Included Studies. Y = yes; N = no; CD = cannot determine; NA = not applicable; NR = not reported.

Study	Study design	1	2	3	4	5	6	7	8	9	10	11	12	13	14	Quality
Fullmer (2022) [42]	Cross-Sectional Retrospective	Y	Y	NR	Y	N	Y	Y	Y	Y	N	Y	CD	NA	Y	Fair
Bhandari (2021) [43]	Non-Randomised Control Trial	Y	Y	Y	Y	Y	Y	Y	Y	Y	Y	Y	NR	Y	Y	Good
Aristizabal (2021) [44]	Cross-Sectional Retrospective	Y	Y	NR	Y	CD	Y	Y	Y	Y	Y	Y	NR	CD	Y	Fair
Juhász (2020) [45]	Randomized Controlled Trial	Y	N	Y	Y	Y	Y	Y	Y	Y	Y	Y	Y	NR	Y	Good
Gurlek Gokcebay (2018) [46]	Prospective Observational Cohort	Y	Y	CD	Y	N	Y	Y	Y	Y	Y	Y	NR	CD	N	Good
Mohan (2016) [47]	Case Control	Y	Y	Y	Y	Y	Y	NA	NR	Y	Y	Y	Y	NA	NA	Fair
Iniesta (2016) [48]	Prospective Cohort	Y	Y	Y	Y	N	Y	Y	Y	Y	Y	NR	N	Y	Y	Good
Helou (2014) [49]	Cross-Sectional	Y	Y	Y	Y	NR	Y	Y	Y	Y	Y	Y	Y	Y	Y	Good
Nematollahi (2024) [50]	Observational Cohort	Y	Y	Y	Y	N	Y	Y	Y	Y	N	NA	N	Y	Y	Good
Malecka (2022) [51]	Cross-Sectional	Y	Y	Y	Y	N	Y	Y	Y	Y	N	Y	NR	NA	N	Good
Song (2022) [52]	Observational Cohort	Y	Y	Y	Y	N	Y	Y	NA	Y	N	NA	Y	Y	Y	Good
Sherief (2021) [53]	Cross-Sectional	Y	Y	Y	Y	N	Y	Y	NA	Y	Y	NA	Y	Y	Y	Good
Maddheshiya (2021) [54]	Observational Cohort	Y	Y	Y	Y	N	Y	Y	NA	Y	Y	NA	Y	Y	Y	Good
Norouzi (2021) [55]	Case Control	Y	Y	Y	Y	N	NR	N	Y	Y	Y	Y	Y	NA	NA	Fair
Bhattacharya (2020) [56]	Prospective Observational Cohort	Y	Y	NR	Y	N	Y	Y	Y	Y	Y	Y	NR	Y	N	Good
Oosterom (2019) [40]	Prospective Observational Cohort	Y	Y	NR	Y	N	Y	Y	Y	Y	Y	Y	NR	CD	Y	Good
Orgel (2017) [57]	Randomized Control Study	Y	Y	NR	N	N	Y	NR	Y	Y	Y	Y	Y	Y	Y	Fair
Jackmann (2020) [58]	Cross-Sectional	Y	Y	Y	Y	N	Y	Y	Y	Y	N	Y	N	NR	Y	Good

Cook (2014) [59]	Case Report	Y	Y	Y	Y	Y	Y	Y	Y	NA	NA	NA	NA	NA	NA	Fair
Izurieta (2023) [60]	Retrospective Observational Cohort	Y	Y	NR	Y	Y	Y	Y	Y	Y	N	Y	NR	NA	N	Good
